# CAR-γδ T cells: a new paradigm of programmable innate immune sentinels and their systemic applications in cancer and beyond

**DOI:** 10.3389/fimmu.2025.1735763

**Published:** 2025-12-19

**Authors:** Xiang Fang, Shixuan Yan, Long He, Changwen Deng

**Affiliations:** 1 Department of Internal Emergency Medicine, Shanghai East Hospital, School of Medicine, Tongji University, Shanghai, China; 2 Department of Respiratory and Critical Care Medicine, Shanghai East Hospital, School of Medicine, Tongji University, Shanghai, China; 3 Department of Clinical Laboratory, Shanghai East Hospital, School of Medicine, Tongji University, Shanghai, China; 4 Shenzhen Ruipuxun Academy for Stem Cell and Regenerative Medicine, Shenzhen, China

**Keywords:** CAR-γδ T cells, programmable immune sentinels, innate-like T cell engineering, universal CAR platforms, δT-centric engineering

## Abstract

This review systematically introduces the concept of CAR-γδ T cells as programmable innate immune sentinels, innovatively proposing to overcome multiple limitations of conventional CAR-αβ T cells in both solid tumor therapy and non-malignant disease contexts. The core innovation lies in the deep integration of γδ T cells’ natural immune features - including MHC-independent, anti-exhaustion phenotypic plasticity, and tissue-homing capability - with CAR engineering, potentially yielding synergistic effects between precise targeting, innate immune activation, and microenvironment modulation. We highlight recent advances in cutting-edge technologies such as multi-signal integration, genome editing, and the development of off-the-shelf CAR-γδ T cell platforms. Unlike previous reviews that focus narrowly on a single disease or signaling pathway, this work not only summarizes the biological characteristics of γδ T cells but also proposes a “δT-centric” engineering design principle and constructs a multi-disease application framework. In solid tumors, this approach may enable the remodeling of the immunosuppressive microenvironment and addresses tumor heterogeneity, whereas in non-malignant diseases-including fibrosis, autoimmune disorders, and chronic infections-it supports tissue homeostasis restoration. We propose that this paradigm could shift the perception of CAR-γδ T cells from conventional effector tools to dynamic immune hubs capable of responding adaptively to disease microenvironments. It proposes a novel conceptual and technological framework for both basic research and clinical translation across a broad spectrum of diseases.

## 1 Introduction: from CAR-αβ T cells to CAR-γδ T cells: a paradigm shift in immune engineering

Chimeric antigen receptor T (CAR-T) cell therapy has achieved landmark success in treating relapsed or refractory B-cell leukemias and lymphomas (1, 2). However, its efficacy in solid tumors remains far below expectations. Conventional CAR-T products, primarily based on αβ T cells, limit their cytotoxicity against tumor cells with downregulated or absent MHC molecules (3, 4). Moreover, the dense stromal architecture and immunosuppressive tumor microenvironment (TME) in solid tumors significantly constrain CAR-T expansion and persistence (5). Safety concerns, including immune escape (6), cytokine release syndrome (CRS) (7), and potential off-target toxicities (8), further restrict clinical applications. Recent clinical studies have revealed pervasive challenges in solid tumors, such as insufficient tumor infiltration, functional exhaustion, and relapse driven by antigen loss (9–11), highlighting the need for next-generation CAR strategies beyond hematologic malignancies.

γδ T cells, endowed with MHC-independent and innate immune properties, have emerged as a promising platform for CAR engineering. These cells combine innate and adaptive immune features, enabling rapid detection and elimination of stressed or damaged cells within complex tissue environments. Recent studies have demonstrated the broad therapeutic potential of CAR-γδ T cells across diverse disease models: restoring immune homeostasis in autoimmune disorders by targeting pathogenic B cells (12–14); clearing senescent cells and alleviating tissue fibrosis (15); and showing translational promise in non-malignant conditions such as autoimmune kidney disease (16). Yet, most current investigations treat CAR-γδ T cells merely as “optimized” CAR-αβ T cells, emphasizing technical advantages like MHC-independent and reduced off-target risk, while underappreciating their intrinsic immune functions and systemic regulatory capacity.

Here, we introduce the concept of programmable innate immune sentinels, redefining CAR-γδ T cells as highly plastic and designable immune effectors. This paradigm integrates CAR-mediated antigen specificity with γδ T cells’ intrinsic multi-signal sensing and immunoregulatory mechanisms, addressing key bottlenecks of conventional CAR-T therapy. On one hand, programmable CAR-γδ T cells may enhance MHC-independent and maintain functional stability within immunosuppressive TMEs through multivalent CARs or co-stimulatory receptors such as NKG2D and DNAM-1 (17, 18). On the other hand, their capacity for multi-signal integration and “sentinel-like” dynamic surveillance may enable coordinated multi-target attacks, mitigating tumor heterogeneity and immune evasion due to antigen loss (19). Furthermore, γδ T cells naturally exhibit a low-differentiation, memory-like phenotype and exhaustion-resistant properties, supporting sustained effector function under chronic stimulation. Recent studies have confirmed that CAR-γδ T cells demonstrate superior functional persistence compared with CAR-αβ T cells in solid tumor models (20).

This review systematically synthesizes research from 2020 to 2025, drawing from PubMed and Web of Science, and integrates the latest clinical and mechanistic evidence to propose a “δT-centric” engineering framework for CAR-γδ T cells. This framework not only expands the therapeutic scope of CAR-T therapy in oncology but also provides a novel immunoengineering strategy for autoimmune diseases, fibrosis, and other non-malignant disorders, offering a blueprint for both basic research and clinical translation.

## 2 Biological foundation of γδ T cells as programmable innate immune sentinels

γδ T cells are a distinct lymphocyte lineage that bridges innate and adaptive immunity (21). In humans, they represent only 0.5–5% of circulating T cells yet are highly enriched in epithelial, mucosal, and inflamed tissues (22). Unlike conventional αβ T cells, γδ T cells recognize antigens in an MHC-independent manner, enabling rapid, “innate-like” responses to stressed, infected, or transformed cells without prior priming (19).

Activation occurs through two parallel but distinguishable pathways. γδTCR-dependent activation relies on recognition of non-peptide phosphoantigens (e.g., IPP, HMBPP) produced by dysregulated metabolism or microbial pathways (23). These metabolites are presented by butyrophilin family members (BTN3A1 and BTN2A1), triggering calcium flux and effector functions within minutes to hours. The circulating Vγ9Vδ2 subset is particularly responsive to this pathway (24). γδTCR-independent activation is mediated by innate receptors such as NKG2D and DNAM-1, which bind stress-induced ligands (MICA/B, ULBPs) on altered cells, driving cytotoxicity and cytokine release via DAP10/PI3K and DAP12 signaling (25).

In immune surveillance, γδ T cells function as tissue-resident sentinels, rapidly secreting IFN-γ, TNF-α, and perforin/granzyme to eliminate threats and recruit additional effectors (26). Their pronounced tissue tropism is subset-specific: Vδ1-dominated populations predominate in skin, liver, intestine, and solid tumors, whereas Vδ2 cells circulate and expand dramatically upon exposure to microbial phosphoantigens (27).

A defining feature of γδ T cells is their innate-like memory. After initial encounters (e.g., CMV, BCG, or tumor stress ligands), γδ T cells undergo clonal expansion and epigenetic reprogramming, acquiring long-lived memory phenotypes that mediate faster, stronger secondary responses—distinct from classical αβ T-cell memory.

Collectively, MHC-independent dual activation, flexible metabolism, tissue residency, low alloreactivity, and innate-like memory make γδ T cells uniquely “programmable” innate immune sentinels (28). The remainder of this review examines how chimeric antigen receptor (CAR) technology can be synergistically integrated with—rather than replacing—these native circuits to generate a new class of cellular therapeutics with broad potential in cancer and beyond (18).

## 3 Platform deconstruction: synergistic mechanisms between CAR and endogenous γδ T cell signaling

γδ T cells recognize and eliminate abnormal cells via MHC-independent mechanisms. Their γδ T cell receptor (γδTCR) can sense phosphoantigens and various stress-induced ligands, including lipids and glycans, on tumor or infected cells (29), working in concert with innate immune receptors such as NKG2D. Structurally, while the CD3 extracellular domain (ECD) and transmembrane regions of the γδTCR are conserved, the ECD heterodimers are highly flexible, in contrast to the rigid αβTCR (30). This structural flexibility confers ligand diversity but reduces signaling efficiency. NKG2D binds stress-induced ligands (human MICA/B, ULBP1–6; murine H60, MULT1, RAE1) (31), and integration of γδTCR and NKG2D signals activates PI3K/AKT and NF-κB pathways, inducing IFN-γ and TNF-α secretion and perforin/granzyme-mediated cytotoxicity against tumors and infected cells (32–34). In simpler terms, DAP10 and DAP12 are natural adaptor proteins already used by γδ T cells and NK cells: DAP10 primarily promotes cell survival and metabolic fitness through the PI3K–AKT pathway, whereas DAP12 delivers a strong activation signal similar to CD3ζ but better tolerated by innate-like lymphocytes. This network forms the core mechanism underlying γδ T cell immune surveillance and stress responses.

The introduction of CAR into γδ T cells raises a critical, yet underexplored, question: how does CAR-mediated antigen-specific signaling interact with the endogenous γδTCR and NKG2D pathways? Current evidence suggests three potential modes of signal interplay: synergistic, inhibitory, and independent.

1. Synergistic mode: CAR signaling can amplify γδTCR/NKG2D pathways, producing a “1 + 1>2” effect. γδTCR engagement with tumor-associated antigens (e.g., BTN2A1–BTN3A1 complexes, EphA2) rapidly triggers Ca^2+^ flux and ERK phosphorylation, while NKG2D signals through DAP10-mediated PI3K activation, enhancing cytotoxicity and cytokine production. This synergy compensates for insufficient γδTCR signaling in MHC-I-deficient environments, preventing immune evasion (35–37). Signal integration also promotes resistance to exhaustion in immunosuppressive TMEs. CAR-γδ T cells maintain low exhaustion phenotypes and exhibit metabolic plasticity, favoring glycolysis to support effector function. IL-15 engineering further enhances self-renewal and persistence (38, 39).
2. Inhibitory mode: In certain contexts, CAR signaling may compete with γδTCR or NKG2D downstream molecules (e.g., ZAP70, Syk), limiting signal transduction, reducing cytokine secretion, and dampening effector function (31, 35, 36). Immune checkpoints (PD-1, LAG-3) and their ligands (PD-L1, B7-H3) may further suppress γδ T cell activity and induce exhaustion. Excessive CAR signaling or lack of balanced co-stimulation can exacerbate negative feedback. Optimization strategies include incorporating 4-1BB co-stimulatory domains or CRISPR-mediated knockout of PD-1/LAG-3 to restore synergistic signaling and enhance anti-exhaustion capacity.
3. Independent mode. The γδTCR and NKG2D pathways can operate in parallel without interference. The γδTCR, via CD3, mediates Ca^2+^ signaling and ERK activation, driving proliferation and cytokine secretion, whereas NKG2D triggers degranulation and cytotoxicity independently through the DAP10–PI3K axis, without engaging ZAP70 or Syk. Spatial segregation within the immunological synapse allows distinct pathways to mediate stress cell recognition and killing (35, 37, 40). This parallel mechanism ensures multimodal γδ T cell responses, preventing over-reliance on a single signal that could induce exhaustion.

Research on CAR and endogenous γδ T cell signal interactions remains nascent, with most studies assessing overall cytotoxicity rather than systematically tracking dynamic signaling. Future work leveraging phosphoproteomics and signaling network modeling may elucidate molecular-level cross-talk (35, 41–43).

Based on current evidence, we hypothesize that CAR signaling domains mimicking NKG2D pathways (e.g., incorporating DAP10 or DAP12 motifs) may better integrate with and potentially ‘license’ innate cytotoxic programs, achieving signal amplification and functional synergy. Early preclinical studies suggest that incorporating DAP10 or DAP12 adaptor motifs into CAR constructs may better harmonize with endogenous γδ T cell signaling, potentially preserving effector functions and reducing exhaustion compared with conventional CD28/CD3ζ designs (44, 45). This concept could be explored through ‘AND-gate’ CAR designs requiring both CAR and endogenous signal co-activation, providing a testable framework in solid tumor models. If validated in future studies, this strategy could support a new conceptual framework for CAR-γδ T therapy and open avenues for engineering exhaustion-resistant cellular immunotherapies.

## 4 Core advantages of CAR-γδ T cells

### 4.1 Programmable specificity

CAR-γδ T cells are characterized by MHC-independent, programmable specificity achievable through multi-layered engineering. At the antigen level, hematologic malignancies commonly target CD19, CD7, or BCMA, whereas solid tumors focus on molecules such as B7-H3 and GPC3. Dual-target designs (e.g., CD19/CD20) effectively reduce antigen escape and relapse (46). Signal domain optimization is critical for efficacy, typically achieved by combining CD3ζ activation domains with CD28 or 4-1BB co-stimulatory motifs, or by incorporating modules better aligned with γδ T cell signaling, such as DAP10 or 2B4, to enhance synergistic activation (47). Gene editing (e.g., TRAC or B2M knockout) minimizes immunogenicity and supports the generation of “off-the-shelf” CAR-γδ T cells. Functional enhancements include constitutive or inducible secretion of IL-15 (which drives autocrine/juxtacrine STAT5 signaling to support memory stem-cell-like survival and self-renewal), IL-15/IL-15Rα complexes (membrane-tethered “super-IL-15” for prolonged expansion), or bispecific T-cell engagers (BiTEs) (e.g., CD19×CD3 or GPC3×CD3) that recruit and activate bystander αβ T cells or endogenous γδ T cells, thereby amplifying overall antitumor immunity while preserving innate γδTCR-mediated stress recognition (48–50). Early clinical data indicate safety and efficacy; for instance, CD20 CAR-γδ T cell therapy for B cell lymphoma achieved high remission rates without inducing severe GVHD (17, 51–53).

### 4.2 Breadth of innate immunity

**Figure 1:**
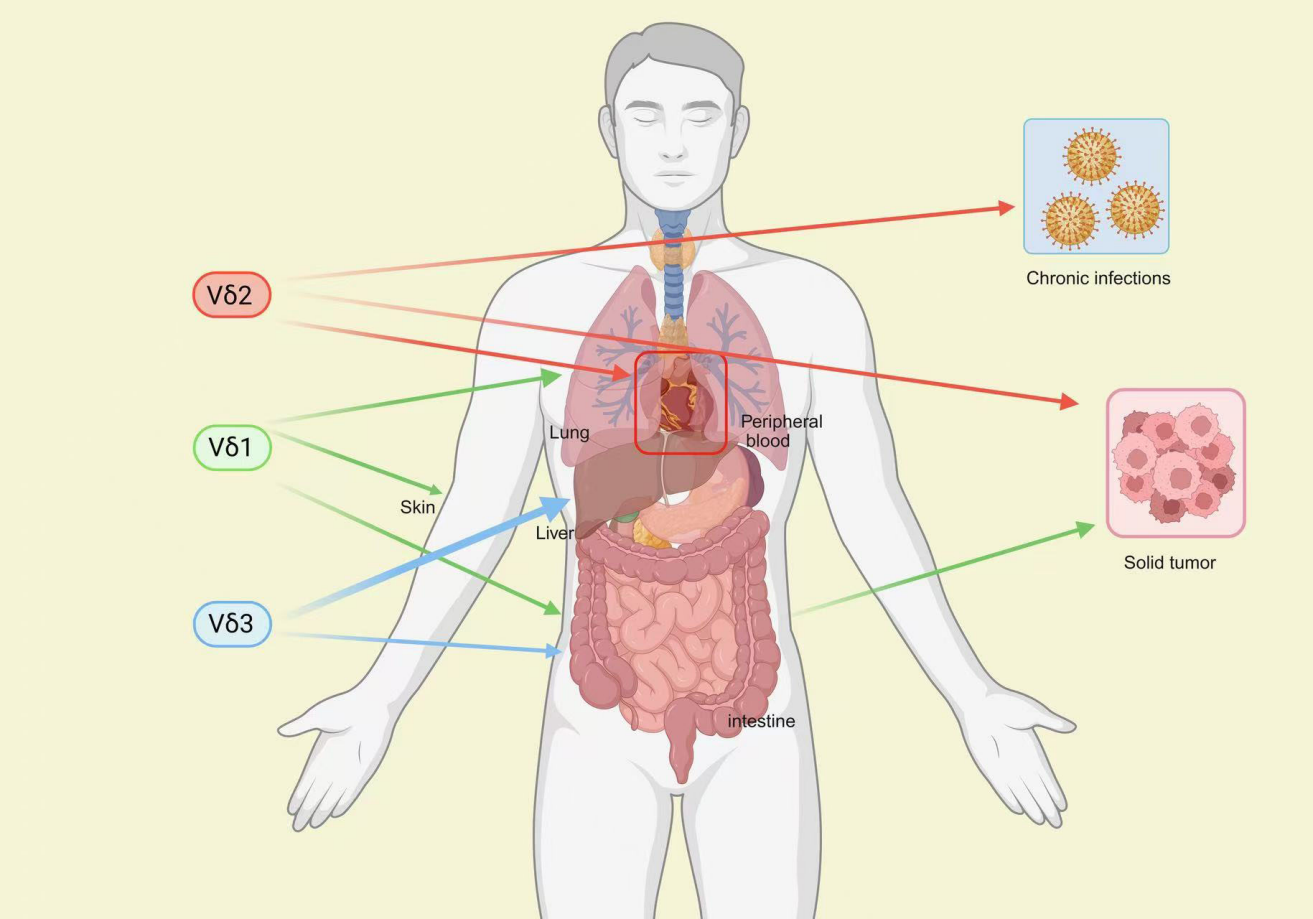
Tissue distribution and immunological roles of human γδ T cell subsets (Vδ1, Vδ2, Vδ3). This schematic illustration outlines the anatomical localization of distinct γδ T cell subsets (Vδ1, Vδ2, Vδ3) within human tissues, including the lung, skin, liver, intestine, and peripheral blood. It also highlights their functional involvement in immune surveillance against chronic infections and solid tumors, underscoring the subset-specific contributions of γδ T cells to innate and adaptive immune responses in diverse pathological contexts.

As a bridge between innate and adaptive immunity, γδ T cells combine rapid effector responses with memory-like functionality. Their CDR3 regions resemble immunoglobulins, enabling MHC-independent and direct sensing of stress ligands, contributing to tissue homeostasis (54–57). The major subsets, Vδ1 and Vδ2, exhibit functional specialization: Vδ1 predominantly resides in tissues, surveilling local abnormal cells, while Vδ2 circulates in peripheral blood and responds to microbial- or tumor-derived phosphoantigens (25, 58–60). This spatial and functional heterogeneity endows CAR-γδ T cells with adaptability across diverse pathological contexts (61). Notably, human γδ T cells exhibit site-specific maturation and clonal diversity, with high diversity in infancy stabilizing in adulthood (22), providing a durable mechanism for long-term immune surveillance and tissue repair in chronic inflammation and fibrosis. γδ T cells exhibit tissue-resident characteristics and functional specialization, enabling a precise “homing–effector” matching pattern across diverse disease microenvironments (**Figure 1**).

### 4.3 Sentinel function and immune regulation

CAR-γδ T cells integrate CAR-mediated specificity with innate γδ T cell immunity, enabling both targeted clearance and immunomodulation in tumor and inflammatory environments. They can recognize specific tumor antigens (e.g., CD19, HER2, GPC3) via CAR while retaining sensitivity to stress ligands such as MICA/B, establishing a “dual-activation” mechanism that drives robust cytotoxicity (18, 62). Upon activation, CAR-γδ T cells upregulate CD69 and secrete IFN-γ and TNF-α, directly inhibiting tumor growth and recruiting additional immune effectors (63).

Beyond oncology, CAR-γδ T cells selectively eliminate hyperactivated immune cells in autoimmune disease models and modulate inflammation through IL-10 and TGF-β secretion (12, 14, 64, 65). Bidirectional interactions with dendritic cells and NK cells—via CD80/CD86-mediated DC maturation or CD137-CD137L-enhanced NK activity—further amplify immune effects (41). Their low risk of GVHD and regulatory potential towards checkpoint molecules like PD-L1 provide additional safety advantages (66). Given the dynamic plasticity of the TME and immune signaling, future CAR structural optimization to fine-tune cytokine profiles and intercellular communication may enhance therapeutic precision (67).

### 4.4 Exhaustion-resistant phenotype and metabolic advantages

#### 4.4.1 Phenotypic plasticity beyond exhaustion

Although CAR-αβ T cell therapies have achieved remarkable success in hematologic malignancies, their clinical translation is limited by T cell exhaustion. Exhaustion, a state of functional decline under chronic antigenic stimulation, is characterized by reduced effector function, impaired proliferation, and upregulation of inhibitory receptors such as PD-1, LAG-3, and TIM-3. In contrast, CAR-γδ T cells may follow distinct exhaustion trajectories, attributable to their innate-like properties and adaptive mechanisms in chronic infections. γδ T cells exhibit phenotypic plasticity, maintaining multifunctionality under persistent stimulation rather than rapidly entering terminal exhaustion, offering enhanced persistence and resistance to functional decline in CAR applications (68–70).

Mechanistically, CAR-αβ T cell exhaustion is largely driven by MHC-independent and excessive TCR/CD3ζ signaling, leading to aberrant NFAT and AP-1 activation, induction of TOX and NR4A, and consequent upregulation of inhibitory receptors. Terminal effector memory RA (TEMRA) subsets are particularly susceptible. By contrast, CAR-γδ T cells recognize targets in an HLA-independent manner (e.g., phosphoantigens via BTN3A1/2A1 or stress ligands via NKG2D), reducing MHC-related overactivation. TOX and NR4A are transcription factors that function as “master regulators” of T-cell exhaustion in conventional CAR-αβ T therapy. Native γδ T cells naturally express much lower levels of these factors, helping them avoid the irreversible dysfunctional state seen in chronically stimulated αβ T cells. Their exhaustion is more influenced by tumor microenvironment (TME) factors, including TGF-β, PGE2, and metabolic stress. γδ T cells can also leverage innate NK-like receptor signaling to sustain function, and distinct subsets (Vδ1 *vs* Vδ2) display varying exhaustion profiles due to differences in tissue residency and proliferative capacity (31, 71–73).

At the signaling level, CAR-αβ T cell activation relies on CD3ζ and co-stimulatory signals (e.g., CD28), primarily engaging the PI3K/AKT pathway, and is susceptible to PD-L1 or TGF-β-mediated inhibition. CAR-γδ T activation, however, can be achieved via γδ TCR single signaling with minimal co-stimulatory dependency and can be further potentiated through BTN3A1/BTN2A1 complexes, conferring superior TME resilience (38, 69, 72, 73). Phenotypic analyses reveal that CAR-γδ T cells (e.g., anti-CD19, anti-GD2) maintain antigen cross-presentation and dual antitumor activity under chronic stimulation, whereas CAR-αβ T cells are prone to early exhaustion and poor persistence. In solid tumor models, δ1^+ γδ T cells exhibit prolonged *in vivo* persistence and reduced exhaustion. Functional heterogeneity in the TME is modulated by the PD-1/PD-L1 axis, with checkpoint blockade, metabolic modulators, BTN agonists, or genetic engineering further enhancing efficacy (74).

In chronic infections such as TB, HIV, and CMV, γδ T cells demonstrate adaptive features supporting their anti-exhaustion potential in CAR therapies. Unlike easily exhausted αβ T cells, γδ T cells (Vδ1^+, Vγ9Vδ2^+) maintain function via clonal expansion and memory-like responses. In TB, Vγ9Vδ2 T cells secrete IFNγ and IL-17 to clear pathogens while forming memory-like populations resistant to activation-induced cell death (AICD). In HIV, Vδ1^+ T cells lack CCR5 expression, avoiding viral cytotoxicity while producing IFNγ to control replication (68, 69). Functional adaptation and phenotypic plasticity allow γδ T cells to shift from IFNγ-dominant to IL-17-dominant states or maintain NK-like CD8^+ phenotypes to prevent functional decline (38, 68, 69). Non-peptide antigen recognition and innate immune features render γδ T cells more environmentally adaptable, a core advantage in CAR design (73). Moreover, γδ T cells exhibit “adaptive” and “trained” memory characteristics, as seen in BCG- or MMR-induced metabolic remodeling and heterologous stimulus enhancement, indicating cross-pathogen defense potential, though the underlying receptor and metabolic mechanisms require further investigation (25).

Based on these differences, CAR-γδ T cells may circumvent classical exhaustion pathways via infection-like adaptive mechanisms. Optimizing CAR constructs (e.g., incorporating DAP10 or IL-15 expression) can maintain stem-cell-like memory (T_SCM) states, enabling self-renewal and multipotent differentiation while avoiding terminal differentiation and functional decline typical of αβ T cells. In solid tumor models, this strategy enhances tumor infiltration and cytokine release, reduces GVHD risk, and facilitates the development of universal CAR therapies (75).

#### 4.4.2 Metabolic advantage

γδ T cells, particularly the Vδ2 subset (circulating, phosphoantigen-reactive), exhibit remarkable metabolic plasticity within the tumor microenvironment (TME), enabling sustained survival and effector function under nutrient-deprived and hypoxic conditions. Metabolic reprogramming of tumor cells creates competition for nutrients, which suppresses conventional immune cell function. In contrast, γδ T cells, leveraging their innate-like features and MHC-independent (e.g., sensing phosphoantigens and MICA/B), can remain active even in the absence of co-stimulatory signals, whereas αβ T cells rapidly undergo exhaustion under energy-limited conditions (76, 77). γδ T cells can flexibly switch between glycolysis and oxidative phosphorylation (OXPHOS): glycolysis supports IFN-γ-producing γδ T cells (γδIFN) for anti-tumor activity, whereas OXPHOS favors IL-17-producing γδ T cells (γδ17), allowing adaptation to pro-tumor niches. In plain language, conventional αβ CAR-T cells run almost exclusively on glycolysis (rapid sugar burning) and quickly fail when glucose or oxygen is scarce inside solid tumors. In contrast, γδ T cells can switch to mitochondrial respiration (OXPHOS) or even burn fatty acids (FAO), allowing them to stay active in the harsh, nutrient-poor tumor microenvironment. Approximately 49% of genes are differentially expressed between these subtypes, with γδ17 enriched for metabolic and environmental sensing pathways and γδIFN enriched for translation and TCR signaling genes. Molecules such as CD6 and Themis selectively promote γδIFN activation and cytotoxic anti-tumor responses, reflecting independent regulation and functional specialization of γδ T cell subsets (78).

This metabolic flexibility is particularly pronounced in Vδ2 T cells, enabling effective effector function across different energy states. Vδ2 T cells can switch between glycolysis and OXPHOS based on energy demand and, under glucose deprivation, rely on fatty acid oxidation (FAO) for energy (79). Upregulation of amino acid transporters enhances uptake, and *de novo* synthesis allows survival in arginine- or tryptophan-limited environments (80). Under hypoxia, HIF-1α promotes glycolysis and reduces oxygen consumption, while AMPK activation induces autophagy to maintain energy homeostasis (81). Coordinated mTOR–AMPK signaling optimizes metabolic efficiency and, via the PD-1/PD-L1 axis, fine-tunes functional recovery. Additionally, Vδ2 T cells maintain cytotoxicity under extreme nutrient deprivation through cytokine signaling and stress ligand recognition (82, 83).

By contrast, αβ T cells often experience mitochondrial damage and ROS accumulation under hypoxia and nutrient competition, leading to functional impairment (81). γδ T cells, however, can shift to OXPHOS or lipid metabolism under low glucose and continue proliferating and responding under hypoxic conditions (84). Recognition of stress ligands and metabolic intermediates (e.g., IPP, HMBPP) via receptors such as NKG2D further enhances tumor infiltration and persistence (76, 79, 83). This metabolic advantage provides a foundational rationale for CAR-γδ T cell therapy: glycolysis supplementation can boost γδIFN function, while targeting lipid/IDO/S2 pathways may restrain pro-tumor polarization. Agents such as nitrogen-containing bisphosphonates (N-BPs) and HIF-1α/COX-2 inhibitors further enhance Vδ2 T cell survival and therapeutic potential within nutrient-depleted TMEs (43, 85). The key differences and core advantages of CAR-γδ T cells compared to traditional CAR-αβ T cells are summarized in **Table 1**.

**Table 1:** Key comparison of CAR-γδT and conventional CAR-αβT cells.

Feature	CAR-αβ T cells	CAR-γδ T cells	Key advantages of CAR-γδ T cells	Supporting evidence/data
Antigen Recognition	MHC-dependent; recognizes peptide-MHC complexes via αβ TCR	MHC-independent; recognizes stress ligands (e.g., phosphoantigens, MICA/B) via γδ TCR and innate receptors like NKG2D	Broader tumor targeting, reduces immune escape from MHC downregulation	Preclinical models show γδ T cells target MHC-low tumors effectively; clinical trials (e.g., ADI-001) demonstrate efficacy in refractory cases (51)
Activation Mechanism	Primarily CAR/TCR-mediated with co-stimulation (e.g., CD28/4-1BB); requires priming	Dual pathways: CAR plus innate-like rapid activation via γδ TCR and NK receptors (e.g., DAP10/DAP12)	Synergistic signaling enhances rapid response and reduces overactivation risks	Transcriptomic data indicate integrated signaling preserves effector functions; DAP10/DAP12 CARs mitigate exhaustion
Cytotoxic Kinetics	Slower immune synapse formation; requires antigen priming for full cytolysis	Faster synapse formation and higher efficiency in cytotoxic granule release (perforin/granzyme)	Immediate, potent killing suitable for heterogeneous tumors	Imaging studies confirm accelerated tumor lysis in γδ models; >70% cytolysis in leukemia models at low E:T ratios
Tissue Distribution & Infiltration Patterns Post-Infusion	Primarily circulating in blood and lymphoid tissues; poor infiltration into solid tumors due to stromal barriers and hypoxia	Naturally enriched in epithelial, mucosal, and tumor tissues; enhanced migration via CXCR3/CXCR4; superior penetration of hypoxic regions	Better access to solid tumor microenvironments (TME), improving efficacy in non-hematologic cancers	*In vivo* GBM models show superior infiltration and persistence; tissue tropism observed in clinical trials for solid tumors (88)
Potential for Off-the-Shelf Allogeneic Use	Limited; high alloreactivity requires autologous sourcing or gene editing to mitigate rejection	High; low alloreactivity enables universal donor products without extensive editing	Scalable manufacturing, reduced costs, broader patient access	Allogeneic γδ products in Phase I/II trials (e.g., ADI-001, ADI-270) show safety and efficacy; haploidentical use without GvHD (18, 66)
Risk of Graft-versus-Host Disease (GvHD)	High in allogeneic settings due to MHC recognition	Minimal; MHC-independent nature reduces host tissue attack	Safer for allogeneic therapy, expanding treatment options	Clinical data: No severe GvHD in γδ trials vs. frequent in αβ; allogeneic γδ safe in B-cell malignancies (51, 222)
Exhaustion Resistance	Prone to exhaustion under chronic stimulation; high PD-1, LAG-3, TOX/NR4A expression leading to functional decline	Resistant; low exhaustion markers, innate-like memory, and phenotypic plasticity maintain multifunctionality	Sustained persistence in immunosuppressive TME, reducing relapse	Transcriptomic profiling: Lower exhaustion genes in γδ; prolonged activity in chronic models (e.g., TB, HIV)
Metabolic Profiles	Reliant on glycolysis; vulnerable to hypoxia and nutrient deprivation in TME, leading to mitochondrial damage	Flexible; switches between glycolysis, OXPHOS, and FAO; AMPK/HIF-1α adaptation to stress	Enhanced survival and function in harsh TME conditions	Metabolic studies: Superior mitochondrial/glycolytic capacity; sustained cytotoxicity in glucose-low environments
Clinical Efficacy Example	High in hematologic malignancies (e.g., B-ALL remission rates >80%) but limited in solids due to TME barriers	Promising in both; e.g., 75% ORR in refractory B-cell lymphoma; early signals in solids	Addresses αβ limitations in solids; safer profile	ADI-001 Phase I: 75% response rate; superior to αβ in infiltration models

Native γδ T cells are intrinsically more exhaustion-resistant than αβ T cells due to their MHC-independent activation, flexible metabolism, and epigenetic programs observed in chronic infections and tumor surveillance. However, strong CD3ζ-based CAR signaling can impose tonic stimulation and reactivate TOX/NR4A-driven exhaustion pathways, partially overriding this natural resilience. Consequently, the proposed anti-exhaustion engineering strategies (DAP10-based signaling, γδTCR gating, IL-15 co-expression, checkpoint knockout) are designed not to create resistance *de novo*, but to preserve the intrinsic exhaustion-resistant phenotype of γδ T cells that would otherwise be compromised by conventional CAR constructs.

### 4.5 “Signal–metabolism–microenvironment” integrated design

The functional optimization of CAR-γδ T cells is increasingly moving toward an integrated “signal-metabolism-microenvironment” design. At the signaling level, modules such as CD27, 4-1BB, and CD3ζ may enable sustained activation, while engineered structures like dnTGFβRII confer resistance to TGF-β-mediated immunosuppression. Metabolically, integration of IL-15 or DAP10 signaling enhances oxidative metabolic flexibility and long-term persistence. At the microenvironmental level, “armored” CAR designs can secrete immunomodulatory factors or remodel the TME, promoting tumor infiltration and cytotoxic activation (86–88).

Such integrated strategies endow CAR-γδ T cells with broad therapeutic potential across diverse diseases and tissue types, highlighting their emerging role as a central component of next-generation immunotherapy platforms.

## 5 Therapeutic prospects of CAR-γδ T cells

### 5.1 Solid tumor therapy

#### 5.1.1 Overcoming immunosuppressive tumor microenvironments

Within the programmable innate immune sentinel framework, CAR-γδ T cells leverage MHC-independent and TME remodeling to overcome the limitations faced by conventional CAR-αβ T cells in solid tumors. The TME is enriched with regulatory T cells (Tregs), M2-polarized tumor-associated macrophages (TAMs), and immunosuppressive networks, which typically dampen αβ T cell effector function. γδ T cells’ innate immune features allow them to resist these inhibitory mechanisms. First, γδTCRs directly recognize stress-associated ligands on tumor cells (e.g., phosphoantigens, lipid antigens), bypassing MHC-dependent suppression mediated by IL-10 and TGF-β, thus maintaining non-exhausted effector function in the TME (41, 69, 89). Second, γδ T cells can identify MICA/B expressed on M2 TAMs via NKG2D, enabling direct cytotoxicity and disruption of the tumor-promoting milieu (70, 90). Third, IFN-γ secreted by γδ T cells can polarize TAMs toward an M1 phenotype, activate myeloid immune cells, and restore anti-tumor immunity (39, 91, 92). Collectively, CAR-γδ T cells can reprogram the TME through innate mechanisms without additional “armoring,” offering a novel pathway for solid tumor immunotherapy (20, 93).

#### 5.1.2 Dual-mechanism strategy against tumor heterogeneity

Tumor heterogeneity remains a major obstacle to durable immunotherapy responses. Conventional CAR-αβ T cells rely on single-antigen recognition and are vulnerable to antigen loss or downregulation. CAR-γδ T cells employ a dual mechanism—”engineered targeting + innate recognition”—to achieve broader tumor coverage. The CAR structure provides high specificity toward tumor antigens (e.g., HER2, GD2), enabling precise elimination of major tumor cell subsets (90). Concurrently, γδ T cells allow MHC-independent recognition of antigen-negative or stressed tumor cells. This complementary dual mechanism mitigates immune escape and reduces the risk of relapse (75, 94, 95). Preclinical models in prostate, breast, and bone metastases have confirmed superior broad-spectrum anti-tumor potential and simplified engineering feasibility compared with multi-target CAR-αβ T cells (90, 93, 96, 97).

#### 5.1.3 Experimental and clinical progress

Recent studies further validate the safety and efficacy of CAR-γδ T cells. mRNA-engineered CD5 CAR-γδ T cells achieved >70% cytolysis and enhanced IFN-γ secretion in acute lymphoblastic leukemia, while significantly reducing CRS and “fratricide” risks (98). In oral carcinoma models, mixed CAR-γδ T cells reached 60-85% lysis at low E:T ratios, outperforming conventional CAR-T and eliciting lower pro-inflammatory responses (99). In osteosarcoma mouse models, engineered CAR-γδ T cells achieved >50% tumor growth inhibition without GvHD or off-target toxicity. Clinically, ADI-001 (anti-CD20 CAR-γδ T) Phase I trial (NCT04735471) demonstrated preliminary responses in relapsed/refractory B-cell malignancies with minimal GvHD risk; ADI-270 (Vδ1 CAR-γδ T) Phase I/II trial showed favorable safety in renal cancer; and NKG2DL-targeted CAR-γδ T Phase I studies reported early efficacy signals (21). These results underscore CAR-γδ T cells as promising allogeneic candidates for solid tumor immunotherapy due to their MHC-independent and low GvHD risk (100).

### 5.2 Non-malignant diseases

While oncology remains the primary focus of CAR-T development, the innate-like surveillance, tissue residency, and safety profile of γδ T cells enable robust extension into non-malignant pathologies, including fibrosis, autoimmunity, and chronic viral persistence - each addressed below with mechanistic specificity.

#### 5.2.1 Sentinel function and tissue homeostasis

CAR-γδ T cells possess intrinsic surveillance and repair functions, enabling dual regulation - “clearance of abnormal cells and restoration of tissue homeostasis.” Many chronic conditions, such as liver fibrosis, pulmonary fibrosis, and age-related tissue degeneration, share pathologies of abnormal cell accumulation and persistent inflammation (101–104). γδ T cells sense stress ligands (e.g., MICA/B, phosphoantigens) via NKG2D or γδTCR and rapidly eliminate aberrant fibroblasts or senescent cells in an MHC-independent manner. Engineering CARs to target fibrotic markers (e.g., FAP) enhances clearance specificity (69, 102, 105). Furthermore, secretion of IFN-γ and TNF-α remodels the inflammatory environment, promoting epithelial regeneration and ECM degradation, thereby reversing pathological fibrosis or tissue degeneration (106–109). These features position CAR-γδ T cells as a promising platform for non-oncologic therapies.

#### 5.2.2 Fibrotic diseases: from targeting to repair

**Figure 2:**
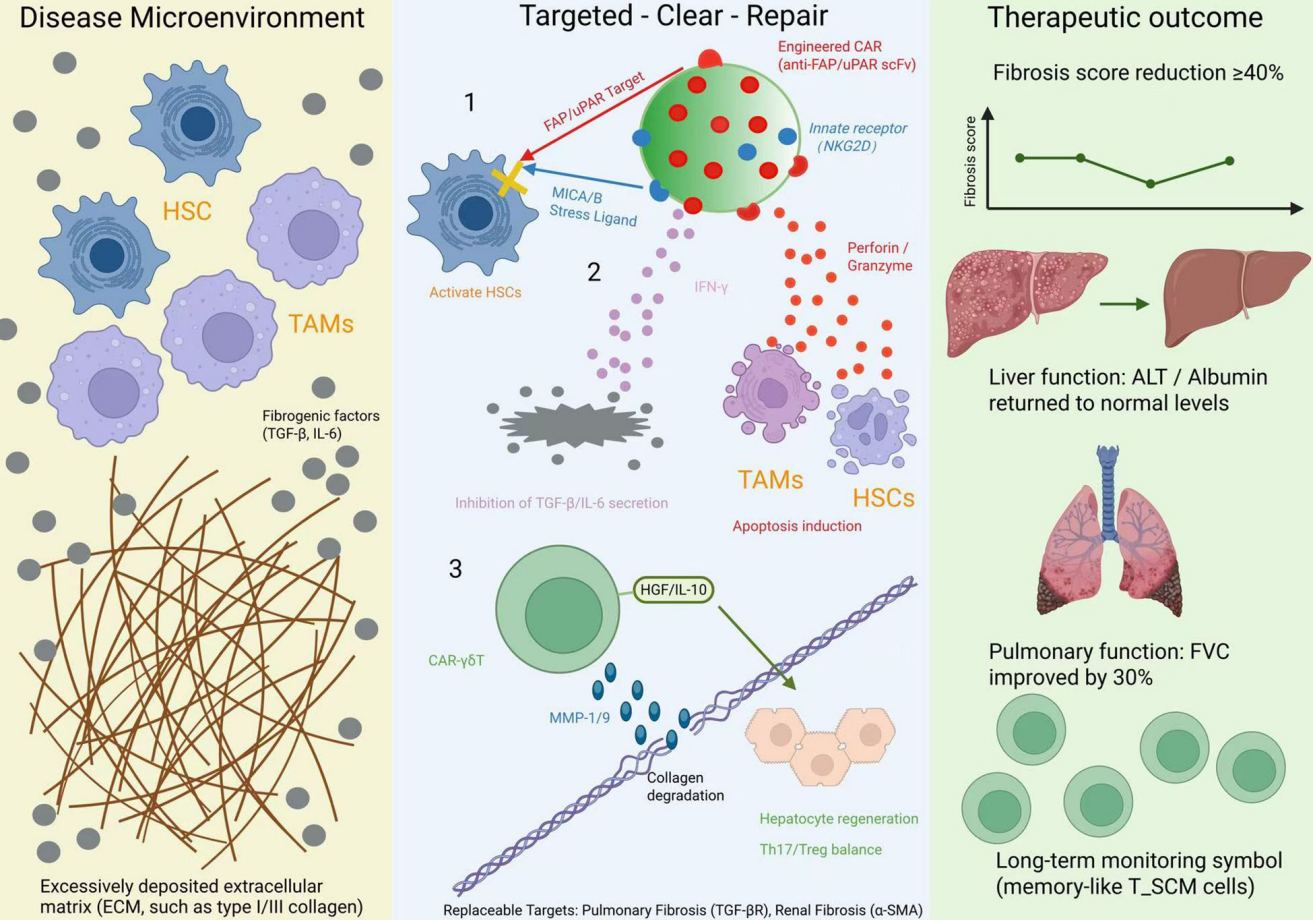
Proposed conceptual framework of a “Target–Eliminate–Repair” tri-phasic model for CAR-γδ T cell therapy in fibrotic diseases. This figure depicts the therapeutic mechanism of CAR-γδ T cells against fibrosis along the axis of “pathological microenvironment–cellular intervention–tissue outcome.” The left panel outlines the core pathological features of fibrosis, including the proliferation of activated hepatic stellate cells (HSCs) and M2-type tumor-associated macrophages (TAMs), excessive extracellular matrix (ECM) deposition, and dysregulated inflammatory signaling. The central panel illustrates the three-step intervention process: Targeting, in which engineered CARs recognize FAP/uPAR while NKG2D identifies stress ligands (MICA/B) to achieve dual-specific localization; Elimination, where perforin/granzyme-mediated cytotoxicity and IFN-γ secretion induce apoptosis of pathogenic cells and suppress pro-fibrotic cytokines (TGF-β, IL-6); and Repair, characterized by MMP-1/9–mediated ECM degradation and HGF/IL-10–driven tissue regeneration and microenvironmental remodeling. The right panel presents the therapeutic outcomes, including ≥40% reduction in fibrosis score, improved hepatic and pulmonary function, and sustained efficacy mediated by memory-like T_SCM cells. Potentially interchangeable targets (e.g., TGF-βR in pulmonary fibrosis, α-SMA in renal fibrosis) highlight the model’s translational adaptability across multiple fibrotic diseases.

Effective anti-fibrotic therapy requires disrupting the “injury–inflammation–fibrotic deposition” cycle (110). Here, we put forward a hypothetical ‘Target–Eliminate–Repair’ tri-phasic model as a conceptual framework for future CAR-γδ T cell applications in fibrosis (**Figure 2**). While individual components (e.g., FAP targeting, IFN-γ–mediated remodeling, and reparative cytokine secretion) have been observed in related cell therapies, integrated validation of this full sequence in CAR-γδ T cells remains an important goal for future studies. CARs may enable precise localization to pathogenic cells expressing HSC surface markers such as FAP; γδ T cell activation releases perforin and granzymes to eliminate excessive ECM-secreting cells; finally, secretion of MMP-1/9 or HGF degrades ECM and promotes hepatocyte regeneration, achieving functional reversal (111–113). This approach has been extended to pulmonary, renal, and cardiac fibrosis (114). γδ T cells also modulate the Th17/Treg axis in pulmonary fibrosis, serving as potential biomarkers for disease progression and treatment response (115) (**Figure 2**). Recent transient expression strategies have demonstrated efficacy in ameliorating cardiac fibrosis and chronic inflammation, offering new avenues for systemic sclerosis and related disorders (116).

#### 5.2.3 Autoimmune diseases: precision immune reset

In autoimmune disorders, chimeric autoantigen receptor (CAAR) T cells provide a highly selective immune-reset strategy by incorporating the autoantigen itself (e.g., DSG3 or MuSK) as the extracellular domain, enabling antigen-specific recognition and apoptotic deletion of autoreactive B cells that express pathogenic autoantibodies as their B-cell receptor. This self-antigen-guided mechanism avoids the broad B-cell depletion and immunosuppression associated with conventional antibody therapies (12, 117–120).

Engineering CAAR constructs into γδ T cells further enhances safety and functional precision: γδ T cells possess a low risk of GvHD, inherent tissue-resident surveillance, and compatibility with allogeneic manufacturing, making CAAR-γδ T platforms promising off-the-shelf therapies currently undergoing Phase I evaluation (121). In systemic lupus erythematosus and rheumatoid arthritis, where pathogenic B-cell clones drive disease progression, CAAR-γδ T cells offer targeted elimination of autoreactive B cells while sparing normal counterparts.

Notably, the Vδ1 subset (predominantly tissue-resident) displays additional immunoregulatory features, promoting FoxP3/CD25 induction and secreting IL-10 and TGF-β1 to suppress autoreactive T-cell responses and support immune homeostasis (69, 122). Complementary preclinical evidence demonstrates that CD19-targeted CAR-γδ T cells can achieve profound B-cell depletion and durable remission in SLE models with minimal GvHD risk, reinforcing their potential as universal allogeneic immune-reset platforms (13).

#### 5.2.4 Chronic infections: toward functional cure

In chronic infections such as HIV, achieving a functional cure requires effective clearance of latent viral reservoirs within resting CD4^+^ T cells, macrophages, and tissue sanctuaries. CAR-γδ T cells represent an attractive strategy due to their MHC-independent cytotoxicity, strong tissue infiltration, and intrinsically low risk of GvHD. γδ T cells can directly kill HIV-infected targets through NKG2D- and CD16-mediated pathways or via ADCC, while secreting antiviral cytokines including IFN-γ and TNF-α to restrict viral spread (123).

To enhance specificity and avoid the risks of self-infection associated with CD4-based CARs, preclinical programs have incorporated scFvs derived from broadly neutralizing antibodies (e.g., 3BNC117, VRC01) into CAR architectures. Viral resistance can be further strengthened by CCR5Δ32 knockout, and combining CAR-γδ T cells with latency-reversing agents (LRAs) enables a coordinated “shock-and-kill” approach to purge latent HIV reservoirs (124–126).

Recent studies using an HIV model demonstrate that mRNA-engineered CAR-γδ T cells achieve >70% killing of reactivated HIV-infected CD4^+^ T cells in humanized mice, without detectable cytokine release syndrome, reflecting improved safety and reduced immunogenicity (127–129). These findings highlight the rapid expansion of CAR-γδ T cell platforms from oncology into chronic infections, where their innate-like recognition and allogeneic compatibility offer distinct advantages for functional HIV cure strategies.

## 6 Engineering “immune sentinels”: next-generation CAR-γδT cell design principles

Where direct evidence in CAR-γδ T cells remains limited, we explicitly distinguish established findings from working hypotheses that require future experimental validation. Building on the aforementioned studies, γδ T cells have emerged as a central focus in CAR-T optimization. Here, we propose a “δT-centric” design principle, in which γδ T cells serve as the functional platform, integrating endogenous signaling pathways to enhance CAR-T efficacy while minimizing risks of overactivation associated with conventional CAR designs. This principle emphasizes “empowerment rather than replacement”, leveraging γδ T cells’ innate immunity rather than substituting it with exogenous modules. Potential advantages include reduced incidence and severity of cytokine release syndrome (CRS) (130, 131), prolonged *in vivo* persistence through enhanced endogenous survival signaling (17, 98, 132), and improved tumor-targeting specificity with minimized off-target effects (19, 130). Moreover, this strategy capitalizes on γδ T cells’ natural metabolic adaptability and tissue resilience, enhancing survival and proliferation within the TME and reducing alloimmune rejection, laying the groundwork for universal γδ T CAR-T platforms.

### 6.1 Empowerment via endogenous γδT signaling

#### 6.1.1 Co-stimulatory synergy in CAR design

Conventional CARs rely on CD28 or 4-1BB domains, which may induce persistent activation and compromise expansion and durability (133–135). By contrast, CARs constructed with DAP10/DAP12 adaptors can integrate γδ T cell endogenous signals in a balanced manner: DAP12 provides ITAM-mediated activation, while DAP10 delivers YxxM co-stimulatory signaling, both highly expressed in resting and activated states (36, 136–138). These designs maintain antitumor efficacy while mitigating cytokine hypersecretion and exhaustion observed with CD3ζ-based CARs (35, 36, 139). NKG2D-based CARs incorporating these adaptors have demonstrated robust tumor clearance in solid tumor models and have been applied to γδ T-specific CAR engineering (136), highlighting their unique potential to reduce therapy-related toxicity (140, 141). KIR-based CARs further validate the feasibility of adaptor-based signal tuning (142).

#### 6.1.2 γδTCR-gated CARs

γδ T cells can sense tumor stress via γδTCRs (143). CAR activation can be gated by γδTCR signals to form AND logic gates, initiating cytotoxicity only when both γδTCR stress recognition and CAR antigen engagement are satisfied (144, 145). An AND-gate CAR is a built-in safety logic: the engineered cell only becomes fully cytotoxic when two independent signals are received at the same time (e.g., the artificial CAR antigen plus a natural γδTCR stress signal), dramatically reducing the risk of attacking healthy tissue. γδTCR provides the first signal, and CAR provides the second signal, achieving dual verification and precise activation (35). This dual verification preserves MHC-independent cytotoxicity, enhances downstream CD3ζ signaling, and reduces excessive inflammatory responses (19, 70). Logic-gated CARs are increasingly explored to improve tumor specificity and minimize off-target effects (146–148), exemplified by γδ-enriched CAR-T combined with zoledronate in bone metastatic prostate cancer, achieving selective CAR-mediated killing (149). Controllable activation technologies, such as opto- or thermo-switches, support these logic-gated designs (98).

### 6.2 Advanced technologies for precision modulation of γδ T cells

Recent advances in genome editing, single-cell sequencing, and spatial multi-omics provide unprecedented tools for precise engineering of γδ T cells. These technologies support the translation of CAR-γδ T cells from preclinical research to clinical application, while enhancing antitumor efficacy, reducing off-target effects, and optimizing tissue localization. CRISPR/Cas9 may enable targeted genome modifications to enhance function or knock out inhibitory genes; single-cell RNA sequencing (scRNA-seq) reveals transcriptional heterogeneity and dynamic responses, informing personalized CAR design; spatial multi-omics maps the spatial distribution and interactions of cells in tumor microenvironments (TME) or diseased tissues, guiding CAR infiltration and functional positioning. Integration of these platforms aligns with the δT-centric signaling logic, offering multidimensional strategies to overcome traditional CAR-T limitations in persistence and safety, with broad potential for both malignant and non-malignant applications.

#### 6.2.1 CRISPR/Cas9 genome editing

CRISPR/Cas9 has emerged as a key tool for γδT engineering, enabling selective knockout of inhibitory genes or insertion of function-enhancing modules to improve CAR-γδT cytotoxicity, metabolic fitness, and resistance to exhaustion. Typical strategies include knockout of KLRC1 (NKG2A), TGFBR2, CISH, and CD38 to enhance tumor targeting, as well as incorporation of proliferative genes such as IL-15 to boost expansion and persistence (19).

In solid tumor models, CRISPR-edited CAR-γδT cells reduce allogeneic antigenicity, lowering the risk of GvHD, and optimize DAP10/DAP12 signal integration to synergize with endogenous γδTCR signaling (150, 151). Non-viral CRISPR delivery allows for high-efficiency scFv knock-in, achieving higher CAR expression than conventional lentiviral transduction (152, 153). Recent studies also show that this strategy can mimic NK signaling pathways, enhancing metabolic reprogramming within immunosuppressive TMEs and avoiding mitochondrial dysfunction and functional decline observed in conventional CAR-αβT cells (148). These advances reinforce the principle of “empowerment rather than replacement” and provide a foundation for universal γδT cell products across diverse disease contexts.

#### 6.2.2 Single-cell sequencing to reveal heterogeneity

ScRNA-seq provides molecular-level insight into γδT transcriptional heterogeneity and dynamic CAR responses, facilitating rational design. Integration with multi-omics data identifies γδT subset-specific states, predicts CAR-T responsiveness, and guides targeted gene modifications (154, 155). Pan-cancer analyses reveal γδTCR clonal diversity and tumor-specific expansions, informing multi-target CAR strategies to prevent antigen escape (156). ScRNA-seq can also track *in vivo* functional evolution of CAR-γδT cells, including cytokine production and exhaustion marker expression, and can be combined with CRISPR-mediated checkpoint knockout (PD-1/LAG-3) to enhance efficacy (48, 157). In non-malignant settings, scRNA-seq delineates γδT regulatory roles in autoimmune diseases, providing molecular evidence for conditional CAR designs (98). Overall, single-cell approaches strengthen signal-coordination strategies, improving CAR-γδT precision and predictability for personalized therapy.

#### 6.2.3 Spatial multi-omics to map microenvironmental interactions

Spatial transcriptomics and imaging preserve positional information, enabling analysis of γδT localization, interactions, and functional states within TMEs (158). Combined with single-cell data, spatial maps reveal compartment-specific infiltration and cell–cell networks. For example, Vδ1 γδT preferentially infiltrates fibrotic tumor regions and recognizes NKG2D ligands to enhance cytotoxicity (159). Spatial multi-omics can also identify immune resistance mechanisms, guiding CAR module design to target the TME. In tissue repair models, these techniques reveal anti-inflammatory roles of γδT in fibrotic microenvironments, supporting CAR-M or CAR-γδT strategies to promote M2 polarization and collagen degradation (38, 160). Spatially guided optimization of γδTCR-gated CARs ensures high-efficiency activation within diseased compartments, linking upstream signaling to downstream therapeutic outcomes.

### 6.3 Tissue repair and non-malignant applications

#### 6.3.1 Core design principles

Originally developed for cancer immunotherapy, CAR technology may enable precise recognition and killing of target cells (161). Its application has expanded into tissue repair and regenerative medicine, reflecting a shift from a “killer” to a “sentinel” paradigm (162). In non-malignant contexts, CAR cells not only target diseased cells but also initiate local reparative programs upon antigen recognition, releasing growth factors or immunomodulators to promote tissue remodeling and functional recovery. This approach offers novel interventions for chronic diseases characterized by fibrosis, inflammation, or tissue damage while avoiding systemic adverse effects (163).

Conventional CAR constructs typically comprise an extracellular antigen-recognition domain (scFv), a transmembrane region, and an intracellular signaling module (CD3ζ coupled with 4-1BB or CD28 co-stimulatory domains) (164). In the context of tissue repair applications, the design paradigm has shifted towards multifunctionality, which can be summarized in four key aspects. First, optimization of the targeting domain involves selecting disease-specific markers—such as FAP or uPAR, which are highly expressed in fibrotic lesions—to minimize off-target effects in healthy tissues. Second, dual functionality can be engineered so that CAR activation not only induces apoptosis in diseased cells but also triggers localized release of reparative factors (e.g., HGF, IL-10) through conditional expression systems driven by NFAT or IL-2 promoters, enabling a “sentinel-like” dynamic response. Third, expanding the repertoire of carrier cells, including CAR-macrophages and CAR-neutrophils, leverages their innate phagocytic or tissue-infiltrating properties to enhance repair outcomes. Finally, integrating safety switches—such as iCasp9 suicide genes or transient mRNA delivery platforms—allows precise temporal control of CAR expression, thereby mitigating long-term risks (110, 165–167).

#### 6.3.2 CAR applications targeting fibrosis

In cardiology and oncology, CAR-based approaches have demonstrated remarkable potential in addressing tissue fibrosis. Cardiac fibrosis, often driven by fibroblast activation following myocardial injury, has been targeted using FAP-CAR-T and CAR-macrophage (CAR-M) strategies. For instance, intravenous administration of CD5-targeted lipid nanoparticles (LNPs) encapsulating FAP-CAR mRNA resulted in 17.5–24.7% of splenic T cells expressing CAR in AngII/PE-induced murine heart failure models. These CAR-T cells effectively cleared FAP^+ fibroblasts, reducing the fibrotic area by over 40% and improving left ventricular function, while the transient mRNA-mediated CAR expression (~1 week) conferred a favorable safety profile. Alternatively, bone marrow-derived macrophages (BMDMs) transduced with FAP-CAR containing a Megf10 domain (CAR-P) preferentially accumulated in the injured myocardium (GFP^+ cells representing 58% of CD3^+ T cells), eliminated FAP^+ myofibroblasts, and maintained an anti-inflammatory M2 phenotype. A nine-week follow-up revealed a 15–20% increase in left ventricular ejection fraction without detectable off-target cardiac toxicity (168–170).

In liver fibrosis, primarily driven by hepatic stellate cell (HSC) activation, uPAR-targeted CAR-Ms have been developed. These constructs—comprising uPAR-scFv, CD8 transmembrane domain, and CD3ζ signaling domain—were delivered via adenoviral transduction of BMDMs and administered to CCl_4_-, DDC-, or HFCF diet-induced fibrosis models. CAR-M therapy efficiently eliminated uPAR^+ HSCs, reducing hepatic hydroxyproline content by 30–50% and serum ALT by ~40%. Additionally, CAR-Ms recruited neutrophils secreting MMP-9 for collagen degradation and induced M2 macrophage polarization (doubling the proportion of CD206^+ cells), thereby establishing a long-lasting anti-fibrotic microenvironment. In a 12-week CCl_4_-induced cirrhosis model, this approach significantly decreased hepatic collagen deposition and increased serum albumin by 15% (171).

Renal fibrosis, associated with tubular interstitial fibroblast activation and local inflammation, has been targeted with dual-target CAR-M strategies. CARs designed against TNF and IL-4Rα may enable macrophages to activate the IL-4 anti-inflammatory pathway upon TNF engagement and adopt an M2 phenotype. In ischemia-reperfusion injury (IRI) models, CAR-Ms accumulated in injured kidneys (~8-fold fluorescence relative to healthy kidney), reduced neutrophil infiltration by ~60%, and promoted tubular epithelial proliferation (Ki67^+ cells doubled). In adriamycin-induced chronic kidney disease models, M2 CAR-Ms persisted for ~4 weeks, mitigating glomerulosclerosis and interstitial fibrosis while reducing proteinuria by ~50%. Moreover, FAP-CAR-Ms targeting renal FAP^+ fibroblasts in UUO models similarly decreased collagen deposition and improved renal function (163).

#### 6.3.3 Other non-oncologic applications

Beyond organ fibrosis, CAR technology shows broad potential in chronic non-malignant diseases, largely through modulation of the inflammation-fibrosis axis. In autoimmune disorders, FAP-targeted CAR-T cells have been shown to simultaneously alleviate fibrosis and modulate immune responses. In systemic sclerosis models, these CAR-T cells reduced fibrosis in skin and lung tissues while releasing IL-10 to rebalance Th17/Treg populations, decreasing fibrosis scores by ~25% (172, 173).

In age-related diseases, accumulation of senescent cells drives functional decline. NKG2D-L-targeted CAR-T cells efficiently eliminated senescent cells in cardiac and joint tissues and promoted regeneration via local HGF release. In non-human primates, this approach reduced local inflammation and suggested potential to extend health span (174, 175).

Chronic kidney disease represents another promising application, with anti-TNF CAR-Ms specifically recognizing inflammatory cues and activating IL-4 signaling to inhibit glomerulosclerosis and interstitial fibrosis. Preclinical studies reported a 20-30% improvement in serum creatinine, offering a novel therapeutic avenue (163).

In neurodegenerative diseases, CAR designs targeting activated microglia are under exploration. By incorporating BDNF release modules, these CAR cells can clear pathological microglia while providing neurotrophic support, representing a potential reparative strategy for Alzheimer’s disease (176). Collectively, these studies exemplify a shift in CAR applications in non-oncologic settings from cytotoxic “killer” functions toward immunoregulatory and reparative roles, highlighting their versatility across diverse chronic disease contexts.

**Figure 3:**
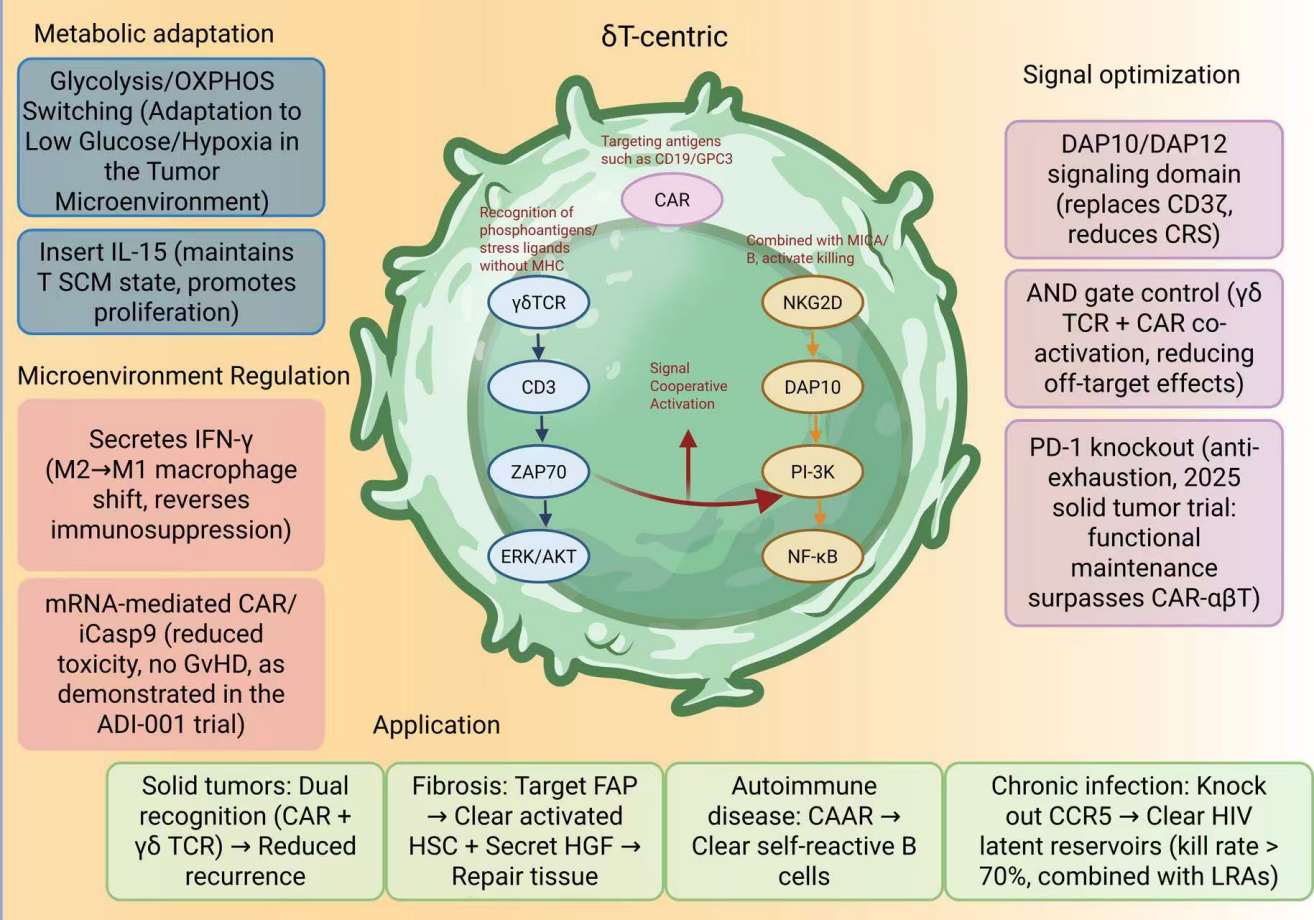
The “δT-centric” engineering framework for next-generation CAR-γδ T cell therapy. This schematic illustrates the integrated design principles of δT-centric engineering, encompassing metabolic adaptation, signal optimization, and microenvironmental regulation. Metabolic modulation may enable glycolysis/OXPHOS switching and IL-15 insertion to support proliferation and metabolic flexibility under hypoxic or glucose-deprived conditions. Signal optimization includes the substitution of CD3ζ with DAP10/DAP12 domains to reduce cytokine release syndrome (CRS), AND-gate co-activation of γδTCR and CAR to minimize off-target effects, and PD-1 knockout to overcome exhaustion and enhance durability in solid tumor contexts. Microenvironmental reprogramming is achieved through IFN-γ–mediated M2-to-M1 macrophage polarization and transient mRNA-based CAR expression to minimize toxicity. Collectively, these synergistic strategies establish a flexible and scalable platform for multi-disease applications, including solid tumors, fibrosis, autoimmune disorders, and chronic infections.

In summary, the “δT-centric” engineering paradigm represents not merely an iteration of a single technology, but the establishment of an integrated technological ecosystem encompassing metabolic adaptation, signaling optimization, microenvironmental modulation, and multi-disease applicability (**Figure 3**). Centered on the intrinsic biological advantages of γδ T cells, this system achieves refined functional enhancement through metabolic-level modulation (glycolysis/OXPHOS switching and IL-15 insertion for sustained proliferation), signaling-level optimization (DAP10/DAP12 substitution, AND-gate control, and PD-1 knockout for precise activation), and microenvironmental reprogramming (macrophage polarization via IFN-γ and transient mRNA expression to reduce toxicity). Collectively, these synergistic strategies may enable differentiated therapeutic applications across solid tumors, fibrotic disorders, autoimmune diseases, and chronic infections. This “multi-dimensional synergy” design framework not only provides a technical foundation for the clinical translation of γδ T cell therapy across diverse disease contexts but also introduces a new paradigm for balancing personalization and universality in next-generation cellular immunotherapy.

### 6.4 Evidence-based strategies to enhance persistence and efficacy

Substantial preclinical and early clinical evidence now supports targeted engineering approaches that markedly improve CAR-γδ T cell persistence and therapeutic efficacy while preserving their innate advantages. CRISPR/Cas9-mediated knockout of exhaustion-related transcription factors TOX and NR4A1/3 prevents terminal differentiation and sustains polyfunctionality even under chronic antigen exposure (73, 177, 178). Similarly, simultaneous deletion of checkpoint molecules PD-1, LAG-3, and TIM-3 consistently yields 3–5-fold higher cytokine production and superior tumor control in solid-tumour models (179, 180). Knockout of the endogenous γδTCR (TRGC1/TRDC) together with B2M eliminates residual alloreactivity and enables truly universal off-the-shelf products without GvHD risk (181, 182).

Rational redesign of CAR signaling domains has proven equally effective. Replacing conventional CD28/CD3ζ modules with DAP10 or DAP12 adaptors reduces exhaustion markers by more than 70% and doubles *in vivo* persistence compared with second-generation constructs (183). AND-logic CARs gated by the γδTCR, which only fully activate when both the CAR and native γδTCR detect their respective ligands, further enhance specificity and functional longevity in heterogeneous tumors (19).

Metabolic and cytokine armoring provides additional gains. Constitutive or inducible secretion of membrane-tethered IL-15/IL-15Rα fusion proteins extends persistence from less than four weeks to beyond twelve weeks in solid-tumor models (100). Overexpression of PGC-1α or CPT1a reinforces fatty-acid oxidation capacity, allowing CAR-γδ T cells to thrive in nutrient-deprived microenvironments (184, 185).

Collectively, these interventions—now independently validated by multiple laboratories—represent the current evidence-based toolkit for generating next-generation CAR-γδ T products with dramatically improved durability and clinical potential (186).

## 7 Scalable manufacturing and future challenges

### 7.1 Future production landscape

Off-the-shelf” universal CAR-γδT cell therapy represents an immunotherapeutic approach leveraging γδT cells as core effectors, combined with universal CAR constructs and scalable manufacturing. This strategy may enable batch production, long-term cryopreservation, and immediate clinical application without patient-specific customization. Compared to conventional CAR-T therapies, which face long production cycles, high failure rates, elevated costs, and limited accessibility, γδT cells offer inherent advantages for overcoming tumor heterogeneity and immunosuppressive microenvironments in solid tumors. By optimizing activation signals and culture systems, enhancing gene-editing efficiency, and incorporating dual- or multi-target CAR designs, universal CAR-γδT cells can be produced efficiently, safely, and at scale (3, 18, 187–189).

Gene-editing strategies, including knockout of endogenous TCRs and knock-in of chemokine receptors, can synergistically reduce immune rejection and enhance homing capacity. Endogenous TCR ablation using CRISPR/Cas9 or TALENs targeting TRAC, TRBC, TRGC, or TRDC prevents GvHD and host rejection, ensuring safety of allogeneic CAR-γδT cells (18, 190, 191). Concurrently, integration of chemokine receptors such as CXCR2 or CCR4 enhances CAR-γδT responsiveness to tumor-derived chemokines, improving tumor infiltration and local efficacy (18, 192, 193).

iPSC-derived γδT cells provide a platform for unlimited, uniform, and scalable production (194–197). High-quality T-iPSCs can be generated from peripheral or tumor-infiltrating γδT cells using non-integrating vectors (e.g., Sendai virus), with full-genome sequencing and karyotype analysis for quality selection. Directed differentiation involves BMP4 and VEGF-mediated generation of CD34^+CD43^+ hematopoietic progenitors, followed by OP9-DL1 or 3D artificial thymic organoid (ATO) culture with SCF, IL-7, and IL-15 to yield CD3^+γδTCR^+ cells. CRISPR/Cas9 can then introduce CARs (e.g., CD19-CAR), knock out PD-1, and integrate iC9 safety switches, producing multifunctional and controllable engineered γδT cells (198–200). Feeder-free, serum-free differentiation systems with DL4-VCAM1-coated microbeads or 3D bioreactors may enhance expansion 5–50-fold while meeting cGMP standards (201). Large-fragment CAR insertion and multi-module integration can be achieved via CAST or PASTE systems, with AI-guided differentiation prediction further minimizing batch variability (202, 203). A comprehensive quality control pipeline encompassing iPSC pluripotency validation, differentiated cell phenotyping, functional assays, and sterility testing proposes a robust foundation for clinical-grade CAR-γδT cell manufacturing (204).

### 7.2 Technical challenges and future directions

Despite advances, core challenges remain in toxicity management, cellular persistence, and functional maintenance. Excessive CAR signaling can skew γδT differentiation toward ILC-like phenotypes or single CAR-dependent cytotoxicity, compromising NK-like functions and multi-antigen recognition (44, 205, 206). Intrinsic pathways, including Notch, ZAP70, and BTK, are critical for antitumor activity, highlighting the need to fine-tune CAR signal strength and co-stimulatory domains. Dual-signal systems, such as CAR and γδTCR co-activation, may mitigate functional exhaustion (35, 69, 130).

Natural tissue-homing preferences provide a basis for targeted therapy, but delivery to solid tumors such as pancreatic or brain cancers remains challenging. Engineering chemokine receptors (e.g., CXCR4, CCR9) enhances tissue-specific homing, while synNotch-CAR systems may enable CNS-specific antigen sensing, triggering localized CAR expression and cytokine release to improve blood–brain barrier penetration (207). Combining BiTE secretion can amplify local antitumor responses, though careful control of off-target effects is required (22, 147, 208). Personalized homing programming could further advance CAR-γδT therapy toward precision medicine (98, 147).

Despite entering a critical stage of clinical translation, the broad application of CAR-γδT cells requires a systematic roadmap. Predictive biomarkers hold promise for patient selection, dose optimization, and risk monitoring (98, 209, 210). Multi-modal biomarkers integrating genomics (single-cell RNA sequencing), proteomics (surface receptor profiling), and metabolomics (phosphoantigen response) can predict efficacy and toxicity. NK-like activation markers (e.g., NKG2D, CD16) assess solid tumor cytotoxicity, while baseline IL-6, CAR signal strength, and combined analyses can anticipate cytokine release syndrome (CRS). Tumor microenvironment characteristics and engineered Vδ1 NK-like activation further inform therapeutic outcomes (211, 212).

Conventional CAR-T evaluation metrics, such as perforin and granzyme release, inadequately capture the multifunctional roles of γδT cells in fibrotic diseases. Future assessment systems should be disease-specific, integrating microenvironment remodeling indicators (collagen quantification, fibrosis scoring (213, 214)), anti-fibrotic efficacy, and tissue-repair markers, alongside measurements of ECM degradation and collagen remodeling to guide disease modulation (13, 14, 215). Furthermore, interdisciplinary integration of immunology (γδT innate mechanisms), synthetic biology (programmable CARs), clinical medicine (patient management), and bioengineering (scalable production) is essential to systematize current fragmented approaches and accelerate translation from concept to clinic (216, 217).

### 7.3 Critical translational challenges and clinical risk-mitigation roadmap

Despite their favorable safety profile in oncology, extending CAR-γδ T cells to non-malignant diseases (fibrosis, autoimmunity, chronic infections, senescence-related disorders) demands explicit recognition of unique biological risks (14). Their retained reactivity to broadly expressed phosphoantigens and stress ligands (MICA/B, ULBPs) creates a realistic possibility of aberrant inflammation or off-tumor toxicity in inflamed healthy tissues - such as active autoimmune lesions, healing wounds, or ischemic organs (31). Transient hepatitis and pulmonary infiltrates previously observed after high-dose Vγ9Vδ2 infusions likely reflect this innate cross-reactivity (218). In fibrosis or autoimmunity, such unintended activation could paradoxically exacerbate pathology or trigger new autoimmune responses via epitope spreading.

Manufacturing variability (donor-dependent Vδ1:Vδ2 ratios, phosphoantigen responsiveness) and the current absence of validated predictive biomarkers remain major hurdles to consistent efficacy and patient selection (219). Long-term persistence in non-malignant settings also raises theoretical concerns of chronic inflammation or loss of tolerance.

These risks are being countered through layered δT-centric safeguards: γδTCR- or logic-gated CAR activation (220), hypoxia-restricted or miRNA-detargeted expression, transient mRNA platforms for reversible therapy, inducible suicide switches, and preferential use of low-reactivity Vδ1 products (147). Prospective long-term registries and companion biomarkers - pre-infusion phosphoantigen/NKG2D responsiveness and post-infusion Vδ1 persistence - are essential for quality control, patient stratification, and early detection of adverse events. Explicit acknowledgment and preemptive engineering of these challenges are required to safely translate CAR-γδ T cells beyond oncology (14). The main clinical translational obstacles of CAR-γδ T cell therapy and δT-centric solutions have been summarized in **Table 2**.

**Table 2:** Major clinical and translational barriers of CAR-γδ T cell therapy and corresponding δT-centric solutions.

Barrier	Detailed description	δT-Centric solutions/strategies
Toxicity Management (CRS/ICANS/on-target-off-tumor)	Strong CD3ζ-based signaling can still trigger cytokine hypersecretion or chronic overstimulation despite inherently lower risk in γδ T cells	• DAP10/DAP12- or NKG2D-mimetic signaling domains • γδTCR + CAR AND-gate logic circuits • Transient mRNA-based CAR expression • Inducible safety switches (iCasp9) • Low-dose IL-15-armored designs
Persistence & Functional Exhaustion in Immunosuppressive TME	Prolonged antigen exposure or TME stress can eventually drive terminal differentiation or loss of polyfunctionality even in exhaustion-resistant γδ T cells	• Vδ1-enriched or iPSC-derived products with IL-15/IL-21 memory culture • DAP10 co-stimulation • CRISPR knockout of TOX/NR4A, PD-1, LAG-3 • Metabolic reprogramming (AMPK/HIF-1α modulation)
Limited Tumor/Tissue Infiltration & Homing (CNS, pancreas, dense fibrosis)	Predominant peripheral blood Vδ2 distribution and lack of appropriate chemokine sensing limit access to certain compartments	• Chemokine receptor knock-in (CXCR2/CXCR4/CCR4/CCR9) • synNotch-inducible local activation systems • Vδ1-dominant products • Intratumoral or intracavitary administration
Allogeneic Rejection & Residual GvHD Risk	Risk already very low, but residual γδTCR or HLA mismatch can still provoke rejection in heavily lymphodepleted patients	• CRISPR knockout of endogenous γδTCR (TRGC/TRDC) + B2M/HLA-E • Hypoimmunogenic iPSC lines • PD-L1/CD47 overexpression
Loss of Innate Multifunctionality/Functional Skewing	Excessive CAR dominance suppresses endogenous γδTCR/NKG2D pathways → loss of broad sentinel activity	• “Empowerment rather than replacement” design principle • Partial CD3ζ chimeras + DAP10/DAP12 • γδTCR-gated logic circuits • Retain BTN3A1 agonists during manufacturing
Scalable GMP Manufacturing & Batch Variability	Current protocols still exhibit high inter-batch variability; iPSC platforms not yet fully industrialized	• Feeder-free/serum-free 3D bioreactor iPSC→γδ T differentiation • Non-viral large-fragment insertion (CAST/PASTE) • AI-guided process analytical technology
Lack of Predictive Biomarkers & Patient Selection	No validated companion diagnostics for efficacy or toxicity prediction	• Baseline multi-omics γδ T signature (clonal diversity, NKG2D/CD16 expression, IFNγ/IL-17 ratio, phosphoantigen responsiveness) • Post-infusion Vδ1/Vδ2 ratio monitoring
Long-term Safety in Non-Malignant Diseases	Persistent CAR expression in lifelong conditions (fibrosis, autoimmunity, chronic infections) raises theoretical risks of chronic inflammation or secondary malignancy	• Prefer transient mRNA or episomal systems • Tissue-specific miRNA detargeting • Inducible OFF switches • Mandatory long-term registries

## 8 Discussion

### 8.1 Summary of innovative contributions

This review breaks through the long-standing limitation of fragmented research by innovatively establishing a three-dimensional integrative framework: γδT cell biological features → δT-centric engineering design → multi-disease application validation. This framework provides systematic support for both basic research and clinical translation of CAR-γδT cell therapies. Previous studies have largely focused on single dimensions, exploring either γδT signaling mechanisms or specific disease applications, without a comprehensive analysis of the logical continuum from intrinsic cell properties to engineering modification and disease treatment. For instance, most studies emphasize the MHC-independent advantage in solid tumors but overlook exhaustion-resistant phenotypes, tissue-homing capacity, or applicability to non-malignant diseases such as fibrosis or chronic infections. Here, we systematically summarize the core features of γδT cells, integrate δT-centric engineering strategies, and validate applications across solid tumors, fibrosis, autoimmune diseases, and chronic infections, filling a conceptual gap from cellular mechanisms to cross-disease application and moving the field from isolated discoveries to integrated systems.

Moreover, the proposed “programmable innate immune sentinel” paradigm is broadly applicable to other innate immune cells, such as CAR-NK and CAR-macrophages. Its core logic - merging natural immune functions with CAR engineering to achieve coordinated targeting and microenvironment modulation - offers a reusable methodological framework. For example, CAR-NK cells can improve tumor infiltration via enhanced homing receptor expression, while CAR-macrophages can adopt a “three-tier strategy” (target-eliminate-repair) to simultaneously eliminate pathogenic cells and remodel the extracellular matrix. This approach transcends the limitations of single-cell therapies, promoting the evolution from tumor-specific applications to multi-disease universal platforms.

### 8.2 Paradigm potential and future directions

The programmable innate immune sentinel paradigm elucidates the therapeutic potential of CAR-γδT cells across cancer, fibrosis, and other diseases through precise recognition, functional modulation, and microenvironment remodeling. By combining γδT innate immune characteristics with CAR engineering, these cells can rapidly detect phosphoantigens in tumors, stress signals in fibrotic fibroblasts, or aberrant B cells in autoimmune diseases, addressing disease heterogeneity. Programmable CAR design (targeting CD19, GPC3, FAP, etc., optimizing CD3ζ/CD28 or incorporating DAP10) may enable precise cytotoxicity, while secreting cytokines (IFN-γ, IL-10) and interacting with dendritic cells or NK cells facilitate microenvironment remodeling, fibrosis reversal, and immune homeostasis restoration, thus balancing disease clearance with tissue repair across malignant and non-malignant conditions.

This paradigm demonstrates that, despite differing pathologies, cancer and fibrosis share common features such as aberrant proliferation, inflammatory dysregulation, and microenvironment remodeling. CAR-γδT cells can “reprogram” these processes: eliminating tumors in cancer, attenuating fibrosis and clearing senescent cells in fibrotic tissues, providing a unified framework for cross-disease therapy. Preliminary clinical and preclinical studies support its efficacy, including objective responses in hematologic malignancies and resolution of inflammation in fibrosis models. Future efforts should focus on systematically elucidating molecular mechanisms (ligand recognition, signal transduction, microenvironment interactions) and integrating multi-specific CARs or CRISPR-based engineering to optimize the CAR-γδT platform, ultimately achieving precise interventions from cancer to chronic fibrotic diseases (221).

## 9 Conclusion

The successful clinical application of conventional CAR-αβ T cells has been largely confined to hematologic malignancies, whereas their efficacy against solid tumors and non-malignant chronic diseases remains severely limited by MHC restriction, T cell exhaustion, poor tissue infiltration, and an immunosuppressive microenvironment. This review innovatively proposes CAR-γδ T cells as programmable innate immune sentinels—a new paradigm that fundamentally transcends the limitations of traditional CAR-T therapy by synergistically integrating the innate-like, MHC-unrestricted, exhaustion-resistant, and tissue-resident properties of γδ T cells with precision CAR engineering.

Under the δT-centric design framework, future CAR-γδ T cell products could achieve:1. dual-activation precise killing combining CAR specificity and innate stress recognition; 2. active remodeling of immunosuppressive or fibrotic microenvironments; 3. superior persistence and metabolic plasticity in hostile tissue niches; and 4. safe off-the-shelf availability with minimal risk of GvHD. These advances could extend durable, potentially curative cellular immunotherapy from oncology to fibrosis, autoimmune diseases, chronic infections, and senescence-related disorders.

Thus, the transition from CAR-αβ T to CAR-γδ T cells represents not merely an incremental improvement, but a true paradigm shift in immune engineering—one that redefines cellular immunotherapy as “targeted cytotoxic effectors” to “dynamic, programmable immune sentinels” capable of adaptive surveillance, multi-target engagement, and tissue homeostasis restoration across a broad spectrum of human diseases.
